# A172 A CLASSIC CASE OF WHIPPLE'S DISEASE

**DOI:** 10.1093/jcag/gwad061.172

**Published:** 2024-02-14

**Authors:** J Hearn, D Webster, S Carpentier

**Affiliations:** Dalhousie University Faculty of Medicine, Halifax, NS, Canada; Dalhousie University Faculty of Medicine, Halifax, NS, Canada; Dalhousie University Faculty of Medicine, Halifax, NS, Canada

## Abstract

**Background:**

Whipple’s disease is a systemic disorder caused by the Gram-positive bacillus *Tropheryma whippelii*. It is classically defined by the four cardinal clinical manifestations of arthralgias, weight loss, diarrhea and abdominal pain. Classic Whipple’s disease is considered extremely rare, with an estimated global incidence of 30 cases per year, though this may be an underestimate.

**Aims:**

To present a case of classic Whipple’s disease, so as to raise awareness and clinical suspicion for this rare condition.

**Methods:**

We performed a retrospective chart review of a case of classic Whipple’s disease diagnosed in Saint John, NB, in 2020.

**Results:**

A 55-year-old female presented to hospital with intractable weakness and diarrhea. Her history was notable for several years of right leg weakness, myalgias and low-grade fevers. More acutely, she presented to hospital due to worsening secretory diarrhea resulting in 15kg weight loss. Initial investigations were remarkable for an elevated C-reactive protein. The patient was admitted for coordinated evaluation for possible rheumatologic and infectious causes. As part of that workup, colonoscopy was performed.

Colonoscopy revealed an abnormal ileum, with villi described as edematous with a mosaic pattern alternating between erythematous and “white tipped”. On histologic assessment, ileal biopsies showed macrophages with PAS-positive diastase resistant granules suspicious for Whipple’s disease. A follow-up esophagogastroduodenoscopy with duodenal biopsies was thus performed to gain further tissue for confirmatory testing. The duodenal mucosa was also noted to have a mosaic pattern with hypertrophic villi alternating between pale and erythematous in appearance. Duodenal biopsies were taken and sent for PCR testing. This testing was positive for *T. whippelii*, confirming the diagnosis of Whipple’s disease.

The patient was initiated on long-term antibiotic therapy with ceftriaxone transitioning to trimethoprim-sulfamethoxazole prior to discharge. She unfortunately re-presented one month post-discharge with recurrent fevers, malaise and arthralgias. Given suspicion that her disease may be resistant to trimethoprim-sulfamethoxazole, she was transitioned to doxycycline and hydroxychloroquine as second-line therapy. She clinically improved on this regimen, and was able to be de-escalated to long-term doxycycline monotherapy after one year. At a recent follow-up, nearly three years from her initial diagnosis, she was doing well with essentially no ongoing symptoms of her Whipple’s disease.

**Conclusions:**

Whipple’s disease is a rare infection presenting with intractable diarrhea and abdominal discomfort. Our case highlights the excellent clinical response that can be achieved with successful diagnosis and management of this otherwise debilitating disease.

**Funding Agencies:**

None

